# Time Evolution of the Skin–Electrode Interface Impedance under Different Skin Treatments

**DOI:** 10.3390/s21155210

**Published:** 2021-07-31

**Authors:** Brendan B. Murphy, Brittany H. Scheid, Quincy Hendricks, Nicholas V. Apollo, Brian Litt, Flavia Vitale

**Affiliations:** 1 Department of Bioengineering, 240 Skirkanich Hall, University of Pennsylvania, 210 S. 33rd Street, Philadelphia, PA 19104, USA; bremu@seas.upenn.edu (B.B.M.); bscheid@seas.upenn.edu (B.H.S.); quincyh@seas.upenn.edu (Q.H.); Brian.Litt@pennmedicine.upenn.edu (B.L.); 2 Center for Neuroengineering & Therapeutics, 301 Hayden Hall, University of Pennsylvania, 240 S. 33rd Street, Philadelphia, PA 19104, USA; nvapollo@gmail.com; 3 Center for Neurotrauma, Neurodegeneration, and Restoration, Corporal Michael J. Crescenz Veterans Affairs Medical Center, 3900 Woodlawn Ave., Philadelphia, PA 19104, USA; 4 Department of Neurology, University of Pennsylvania, 3400 Spruce Street, Philadelphia, PA 19104, USA; 5 Department of Physical Medicine & Rehabilitation, University of Pennsylvania, 1800 Lombard Street, Philadelphia, PA 19147, USA

**Keywords:** equivalent circuit model, skin–electrode interface, skin impedance, skin treatment, wearable sensors

## Abstract

A low and stable impedance at the skin–electrode interface is key to high-fidelity acquisition of biosignals, both acutely and in the long term. However, recording quality is highly variable due to the complex nature of human skin. Here, we present an experimental and modeling framework to investigate the interfacial impedance behavior, and describe how skin interventions affect its stability over time. To illustrate this approach, we report experimental measurements on the skin–electrode impedance using pre-gelled, clinical-grade electrodes in healthy human subjects recorded over 24 h following four skin treatments: (i) mechanical abrasion, (ii) chemical exfoliation, (iii) microporation, and (iv) no treatment. In the immediate post-treatment period, mechanical abrasion yields the lowest initial impedance, whereas the other treatments provide modest improvement compared to untreated skin. After 24 h, however, the impedance becomes more uniform across all groups (<20 kΩ at 10 Hz). The impedance data are fitted with an equivalent circuit model of the complete skin–electrode interface, clearly identifying skin-level versus electrode-level contributions to the overall impedance. Using this model, we systematically investigate how time and treatment affect the impedance response, and show that removal of the superficial epidermal layers is essential to achieving a low, long-term stable interface impedance.

## 1. Introduction

In recent years, wearable bioelectronic sensors have become increasingly ubiquitous in a wide range of clinical, research, and recreational applications [1,2,3,4]. In the majority of these devices, the recording interface is an electrode placed in direct contact with the skin to allow for transduction of biopotentials from the body to the acquisition and processing systems. Achieving a low-impedance interface between the electrode and the skin is vital to transduce signals with high fidelity, as too high of an impedance increases the thermal noise of the electrodes, thus degrading their signal-to-noise ratio (SNR) [5,6,7]. A low SNR may, in turn, prevent clean recording of muscle activation during electromyography (EMG) studies [8], or may cause morphological changes in electrocardiogram (ECG) signatures that can complicate interpretation [9]. The motion artifact susceptibility of skin-based electrodes may also increase with a higher impedance, further degrading signatures such as the QRS complex in ECG recordings [10,11]. A high impedance electrode may also make it difficult to monitor skin hydration levels over time, which can hinder both sweat-based measurements, and investigations of certain skin diseases [12]. Finally, in long-term monitoring and ambulatory applications, impedance stability is highly desirable, as changes in the electrode response as a result of increased impedance may be detrimental to diagnostic accuracy [13,14]. The issue of maintaining a low-impedance skin–electrode interface to enable clean and meaningful recording of skin-based biopotentials has been discussed in several previous works [7,15,16,17,18].

The majority of surface recording electrodes—especially those used in the clinical setting—are composed of metallic contacts, such as platinum (Pt), gold (Au), or silver/silver chloride (Ag/AgCl) [19]. The transduction of bioelectronic currents is often improved using conductive gels, adhesives, or pastes applied between the skin and the electrode, which lower the interface impedance and mitigate motion artifacts [15,19,20,21,22]. However, a prior study revealed that the noise in gelled electrode recordings originates primarily from the skin-gel interface [7]. While additional factors such as the electrode surface area and the amount of pressure applied to the recording contact can also produce slight variations in impedance and recording quality [20,23], skin properties, rather than electrode properties, have been postulated to have the greatest impact on the interface impedance and its stability over time [4,24]. Indeed, a recent report found that the interface impedance is inversely correlated with skin hydration levels, which can decrease by up to 50% over the course of 2 h, especially if non-breathable materials and electrode geometries are used [12]. Thus, an understanding of the multifaceted nature of the skin–electrode interface and its dynamics has serious implications on the design and engineering of low-noise and long-term stable wearable biotechnologies.

The composition of the outermost skin layer—the stratified epidermis and its uppermost layer, the stratum corneum—is perhaps the largest factor contributing to the variability of the interfacial impedance. The epidermis is a highly complex system with chemical, mechanical, and electrical properties that depend on sex, age, diet, sweat and activity levels, as well as environmental conditions such as temperature and humidity, among others [4,12,17,24]. To moderate some of these sources of variability, skin treatments are often applied before placing the electrode, with the most common intervention being abrasion to remove dead skin, oils, dirt, and other debris from the uppermost layers [17,25,26]. However, abrasion can irritate the skin and cause discomfort to the subject, and the initial effects of abrasive skin treatments do not persist over extended periods of time, since the impedance will naturally decrease once the skin rehydrates with sweat [7,12,22,25,27,28].

To understand the skin–electrode interface, as well as investigate the possible effects of different skin treatments or electrode designs on the overall impedance, a variety of equivalent circuit models have also been proposed in literature [4,22,23,25,29,30,31]. Of these, models that distinguish between phenomena occurring at the electrode level from those happening at the skin level could be particularly useful in guiding electrode design or informing skin pre-treatment approaches, as they allow for separating changes at the physiological level of the skin and body, from material changes that occur at the electrode level. However, very few systematic approaches have been proposed to evaluate the effects of different skin treatments on circuit parameters, or how they may change over time [25,30]. In this work we propose a systematic framework to investigate the electrochemical impedance of the skin–electrode interface over time using standard clinical pre-gelled electrodes. We then fit the collected data to an equivalent circuit model, to illustrate the effect of skin treatments on specific system parameters over time. Specifically, we explore mechanical abrasion, chemical exfoliation, and microporation as skin treatments, and compare these to a no treatment control. We monitor the interfacial impedance under these skin conditions over a 24 h period in 14 healthy human subjects. Fitting the experimental impedance data with our proposed model, we extract equivalent circuit parameters, and show how differences in the impedance response at specific timepoints arise from variations at the skin- or the electrode-level. Our findings underscore the influence of the epidermal layers of skin on the long-term behavior of the impedance, suggesting that while abrasive interventions result in the most significant reduction in the initial impedance, over time the skin–electrode interface equilibrates due to natural sweating. This study establishes a rigorous and generalized approach to investigate the dichotomy between skin-level effects versus electrode-level effects when comparing mechanical and chemical skin interventions. This is also the first study that uses a combined experimental and modeling framework to track and analyze impedance behavior over the course of a full 24 h of electrode wear time.

## 2. Materials and Methods

### 2.1. Experimental Procedures

**Figure 1:**
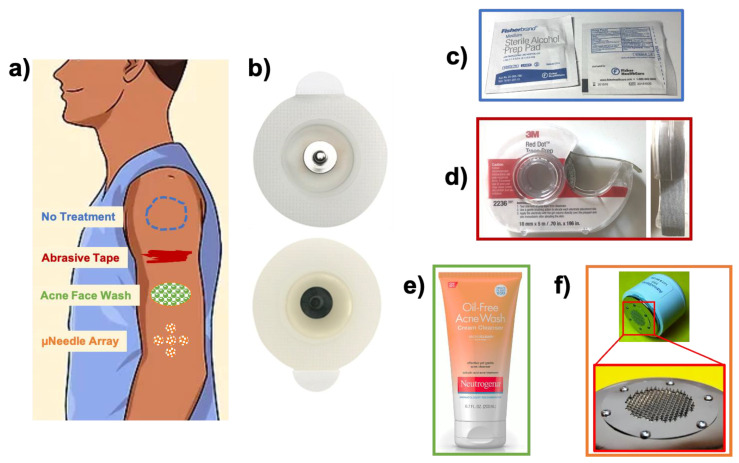
Electrodes and skin treatments used in the present study. (**a**) A cartoon schematic showing application of the four skin treatments to the upper arm. (**b**) Electrodes used in the study: Cleartrace^TM^ 1700-O30 ECG pre-gelled Ag/AgCl disk electrodes (geometric surface area = 5.1 cm^2^, ConMed Co). (**c**–**f**) Skin treatment agents: (**c**) ‘No Treatment’ control (alcohol prep pad for pre-cleaning the skin); (**d**) 3M’s TracePrep^TM^ abrasive tape; (**e**) Neutrogena^®^ Acne Face Wash (with 2% salicylic acid); (**f**) AdminPatch^®^ 0900 microneedle array device.

Fourteen healthy human subjects (6 females, 8 males, avg. age = 28 ± 6 years.) gave written consent to participate in all experimental procedures, following an approved protocol from the Institutional Review Board at the University of Pennsylvania (Protocol No. #833940). Subjects had no history of skin disease or allergy to electrolytic gels or salicylic acid according to self-report. The experimental protocol involved first cleaning the skin of each subject’s upper arms with an alcohol preparation pad (70% isopropyl alcohol (IPA); Fisher Scientific). Then, the skin treatments were applied to each subject’s arms before the recording electrodes were placed (Figure 1). The main skin treatments evaluated were: (i) 3M TracePrep^TM^ abrasive tape (AT), (ii) Neutrogena^®^ Acne Face Wash with 2% salicylic acid (SA), and (iii) the AdminPatch^®^ 0900 microneedle array device from AdminMed nanoBioSciences LLC (µNA). Abrasive tape was selected as it is one of the most commonly used interventions and is known to remove significant portions of the stratum corneum, thus allowing for easier charge-transfer between the more conductive tissue layers of the viable epidermis and the electrode contact [4]. We chose SA because it chemically exfoliates the stratum corneum, but is less abrasive than medical tape [32,33]. We thus hypothesized that SA would be effective in reducing the skin–electrode impedance and improving charge-transfer in a less irritating fashion. The µNA device is an FDA-approved intervention commonly used to deliver drugs and vaccines to the dermis layer of the skin [34,35,36]. It is composed of 85 stainless steel microneedles contained in a 1 cm^2^ circular area, with each needle being 800 µm tall, thus enabling them to penetrate the stratum corneum and viable epidermis layers, though they are shallow enough to avoid blood capillaries and pain receptors located deeper in the dermis [37]. The µNA device typically forms aqueous micropores in the skin surface ≈200 µm in diameter [37], which we hypothesized would allow for readier release of sweat and oils. Thus, we chose microporation with the µNA device to evaluate the effects of deeper penetration on interfacial charge-transfer, compared to more superficial epidermal treatments.

Treatments were applied to skin regions approximately 5 cm^2^ in area, as follows (Figure 1a). A 3-cm-long piece of the TracePrep^TM^ tape was used to abrade a rectangular region of the skin for the AT treatment. For the SA treatment, the SA solution was applied to the skin in a large elliptical region using a Q-tip, and then the wash was left to sit for 2 min, after which the subject was asked to gently rub away the remaining wash using a wet cloth. Finally, for the µNA condition, the AdminPatch^®^ 0900 device was placed in contact with the skin, and pressure was applied while the array was gently rolled back and forth 3x over the skin surface; this same procedure was completed in a cross-pattern to define the overall region where the µNA treatment was applied. After application of all skin treatments, three pre-gelled Cleartrace^TM^ 1700-O30 Diagnostic ECG electrodes (ConMed Corporation; geometric surface area = 5.1 cm^2^; Figure 1b) were placed in a row down the length of the subject’s arm over each treatment region, respectively, and then a fourth electrode was placed over a region of skin that was exposed only to the initial skin cleaning, to provide a “No Treatment” (NT) control condition for each subject. A total of four separate skin conditions were explored for each subject, replicated on both arms, with the level of “harshness” increasing from NT (the gentlest), to µNA, SA, and AT (the harshest). The spatial order in which the treatments were applied along the length of the upper arms was randomized from subject to subject in order to eliminate potential effects from the arm region.

The skin impedance was subsequently measured on both arms of each subject, at three timepoints: (i) after the electrode placement following a 5 min equilibration period (*t*_0_), (ii) at 8 h after placement (*t_mid_*), and (iii) at 24 h after placement (*t_f_*). Before each impedance measurement, two ConMed electrodes were placed on the elbow and over the deltoid as counter and reference electrodes, respectively. The skin impedance was measured via cutaneous electrochemical impedance spectroscopy (EIS), using a Gamry Instruments Interface 1010E potentiostat/galvanostat/ZRA with a 10 mV_rms_ sinusoidal input driving voltage and a 1–10^5^ Hz frequency sweep [38,39,40]. A self-adhering non-woven bandage was wrapped around the subject’s upper arms to protect the electrodes from unconscious everyday movements that might cause displacement or detachment. The wrap was removed for each impedance measurement, and was replaced afterwards.

### 2.2. Data Analysis and Statistics

**Figure 2:**
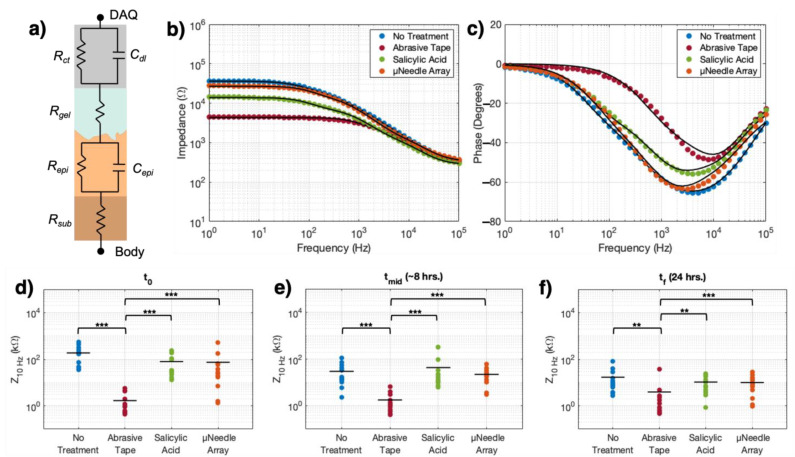
(**a**) Schematic of the equivalent circuit model used for fitting the skin–electrode impedance data. At the electrode level (top three elements): *R_ct_* is the charge-transfer resistance, *C_dl_* is the double-layer capacitance, and *R_gel_* is the gelled electrolyte resistance. At the skin level (bottom three elements), *R_epi_* and *C_epi_* are the epidermal resistance and capacitance, respectively, and *R_sub_* is the resistance of the dermis and subcutaneous fat layers. (**b**,**c**) Representative Bode plots of the impedance (**b**) modulus and (**c**) phase for one subject at the initial timepoint, for all skin treatments. Points represent measured experimental data, while solid black lines denote the fitted curves. (**d**–**f**) 10 Hz impedance values for all subjects, for all skin treatments, at the (**d**) initial (*t*_0_; ANOVA F(14,3) = 68.02, *p* < 1.5 × 10^−15^), (**e**) middle (*t_mid_*~8 h; ANOVA F(10,3) = 19.31, *p* < 5.0 × 10^−4^), and (**f**) final (*t_f_*~24 h; ANOVA F(10,3) = 19.17, *p* < 7.5 × 10^−7^) timepoints. Significance levels: (**) denotes *p* < 0.01, (***) denotes *p* < 0.001.

The equivalent circuit model used for all impedance fitting was built using the EChem Analyst software package (Gamry Instruments), and describes a gelled, metallic electrode interfacing with the epidermis and subcutaneous skin layers (Figure 2a) [4,29]. The total impedance of this equivalent circuit model is expressed by the equation:

(1) $$Z_{tot}=R_{sub}+\left[\frac{R_{epi}}{1+j\omega R_{epi}C_{epi}}\right]+R_{gel}+\left[\frac{R_{ct}}{1+j\omega R_{ct}C_{dl}}\right]\;$$

where *j* is the imaginary unit, ω=2πf is the angular frequency in rad s^−1^, and *f* is the frequency in Hz. This model was chosen over the standard Randles cell [41] to allow for more precise evaluation and comparison of the effects from the electrode versus skin elements. Specifically, the electrode elements include: (i) *R_gel_*, the gelled electrolyte resistance, (ii) *R_ct_*, the charge-transfer resistance and (iii) *C_dl_*, the double-layer capacitance. The representative skin elements include: (i) *R_sub,_* the subcutaneous resistance, (ii) *R_epi_*, the epidermal layer resistance, and (iii) *C_epi_*, the epidermal capacitance. To avoid overfitting and physically implausible values, the parameter space for each circuit element was restricted to specific bounds (Table 1), chosen according to values reported in literature [4,17], and based on considerations of the total impedance magnitude as given in Equation (1). The same boundaries for each model parameter were used across all subjects, treatments, and timepoints. Finally, we normalized the parameter values by the electrode surface area of 5.1 cm^2^, thus providing us with expected values and ranges for these circuit model components which are applicable to a variety of gelled electrode technologies, and are not exclusive to the electrodes used in this study.

For statistical analysis, impedance values for a given treatment and timepoint were averaged across both arms for each subject. Bartlett’s test was then performed on the averaged impedance data, to ensure equal variance across the skin condition groups; if the null hypothesis of Bartlett’s test was rejected, the data were log-transformed to account for a log-normal distribution. Subjects with data points lying 1.5 interquartile ranges outside the extreme quartiles were removed from analysis for that given timepoint. A one-way repeated measures analysis of variance (ANOVA) test was then performed to determine whether there was a significant difference between skin treatments at each timepoint, followed by a pairwise Tukey–Kramer post hoc test. The effect of skin condition on the fitted values of the model parameters was analyzed using three paired rank-sum tests, between the harshest skin treatment group (the AT condition) and the other three groups (NT, SA, and µNA), respectively. The significance level was set at *p* = 0.0167 after a Bonferroni correction, to account for multiple comparisons between skin conditions. All data analysis for the above tests was conducted using custom MATLAB^®^ scripts.

Lastly, using the programming language *R* (The R Foundation) and accompanying package ‘lme4′ [42], we performed a linear mixed-effects analysis to understand the interaction of skin treatment and time on skin- (*R_sub_*, *R_epi_*, and *C_epi_*) and electrode-level (*R_gel,_ R_ct,_ C_dl_*) parameter values. The fixed effects were time and skin treatment (with an interaction term), while the random effects were the intercepts for each subject. We inspected the residuals for any obvious deviations from homoscedasticity or normality, and log-transformed the parameter values if Bartlett’s test failed. Finally, *p*-values were obtained by likelihood ratio testing (LRT) via ANOVA of the linear mixed effects model with the treatment-time interaction term, against a linear mixed effects model without this interaction term. If the interaction was not found to be not significant, the main effect of time on the equivalent circuit parameters was assessed. Otherwise, the simple effect of time for each treatment independently was tested for significance.

## 3. Results

The representative Bode plots for the impedance under each treatment condition at *t*_0_ for one subject are shown in Figure 2b,c. For all subjects, impedance magnitudes across treatment conditions diverged noticeably at frequencies below 5 kHz. The average cutoff frequency (i.e., the frequency at which the phase shift = −45° [43]) for the AT condition was 4.00 ± 2.23 kHz, higher than the average cutoff frequencies of the SA (800 ± 32 Hz), µNA (397 ± 132 Hz), and NT (320 ± 56 Hz) conditions.

Comparison of the average impedance magnitude at the 10 Hz reference frequency [44,45] over time showed that the AT condition maintained a significantly lower impedance than any of the other conditions across the entire duration of the study (Figure 2d–f). At *t*_0_ (Figure 2d), the NT condition had the highest impedance (188.7 ± 163.3 kΩ), while the AT skin treatment impedance was significantly lower than all other treatment types (1.67 ± 0.47 kΩ, *p* < 0.001). The SA and µNA treatments had approximately equivalent impedances (80.0 ± 77.5 kΩ and 74.9 ± 21.3 kΩ, respectively). At *t_mid_* (Figure 2e, ~8 h of contact between the skin and the electrodes), the impedance of the NT, SA and µNA treatments decreased considerably (NT = 29.7 ± 20.2 kΩ, SA = 43.2 ± 23.4 kΩ, µNA = 22.0 ± 15.4 kΩ) while the AT impedance remained essentially unchanged and still significantly lower than all other conditions (1.76 ± 0.77 kΩ, *p* < 0.001). At the 24 h timepoint, *t_f_* (Figure 2f), the average impedance for all skin conditions was < 20 kΩ, although the difference between AT and all other skin treatments was still significant (*p* < 0.01). At each timepoint, the impedance difference between the NT, SA, and µNA treatments was not found to be significant.

**Figure 3:**
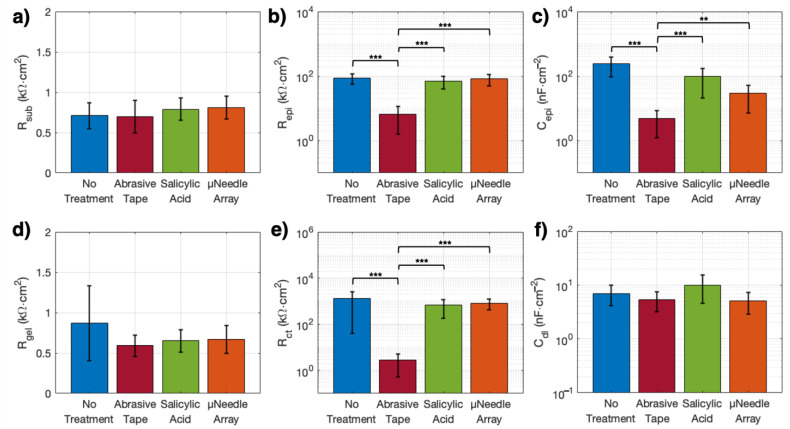
Area-normalized values of the fitted model parameters at *t*_0_. (**a**–**c**) Skin-level results at *t*_0_, for each treatment, including values of the (**a**) subcutaneous resistance, (**b**) epidermal resistance, and (**c**) epidermal capacitance. (**d**–**f**) Electrode-level results at *t*_0_, for each treatment, including values of the (**d**) gelled electrolyte resistance, (**e**) charge-transfer resistance, and (**f**) double-layer capacitance. Significance levels: (**) denotes *p* < 0.0167 and (***) denotes *p* < 0.00167.

The equivalent circuit model provided a consistent fit to the experimental impedance data (goodness of fit between ~2 × 10^−3^ and ~5 × 10^−2^, Pearson’s $\chi^{2}$ test with *df* = 5 degrees of freedom, Table 1). Values of the fitted equivalent circuit parameters for each treatment condition are reported in Table 1 and plotted for timepoint *t*_0_ in Figure 3. At *t*_0_ the parameters describing the epidermal interface, *R_epi_* and *C_epi_*, were in the ranges of 35–86 kΩ cm^2^ and 25–250 nF cm^−2^, respectively, for the three gentler skin conditions (i.e., NT, SA, and µNA), while for AT both values were significantly lower: *R_epi_* = 6.61 ± 5.02 kΩ cm^2^ (*p* < 0.00167, Figure 3b), *C_epi_* = 4.95 ± 3.73 nF cm^−2^ (*p* < 0.00167, Figure 3c). For the circuit elements associated with the electrode interface, the charge-transfer resistance, *R_ct_*, ranged from 0.1–1.5 MΩ cm^2^ in the NT, SA, and µNA conditions, while it was 2.90 ± 2.39 kΩ cm^2^ for the AT treatment (*p* < 0.00167, Figure 3e). In contrast, we found that the resistances of the subcutaneous (*R_sub_*) and gel components (*R_gel_*), as well as the double-layer capacitance, *C_dl_*, were comparable across all skin conditions (Figure 3a,d,f).

**Figure 4:**
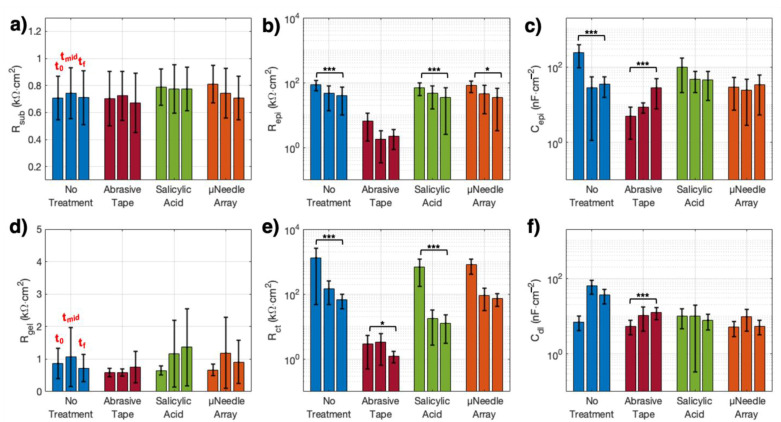
Time evolution of the fitted, area-normalized circuit model parameters over 24 h. (**a**–**c**) Skin-level results for each treatment type, showing time evolution of the values of the (**a**) subcutaneous resistance, (**b**) epidermal resistance, and (**c**) epidermal capacitance. (**d**–**f**) Electrode-level results for each treatment type, showing time evolution of the values of the (**d**) gelled electrolyte resistance, (**e**) charge-transfer resistance, and (**f**) double-layer capacitance. Bars represent means, errorbars are standard deviations (*N* = 14); *t*_0_—initial timepoint, *t_mid_*—study midpoint, 8 h. after skin treatment application, *t_f_*—final timepoint, 24 h. after skin treatment application. Significance levels: (*) denotes *p* < 0.05, (***) denotes *p* < 0.01 (values are reported Table 2).

We also quantified the temporal evolution of the model parameters using the LRT, whereby we determined whether there was a significant interaction between treatment and time for a given parameter (Figure 4 and Table 2). From this analysis, we found significant time-treatment effects at the skin level for *C_epi_* ($\chi^{2}$(6) = 34.2, *p* < 6 × 10^−6^), and at the electrode level for *R_ct_* ($\chi^{2}$(6) = 58.0, *p* < 1 × 10^−10^) and *C_dl_* ($\chi^{2}$(6) = 48.0, *p* < 1 × 10^−8^), whereas there were no significant time-treatment effects for the other parameters. When we subsequently inspected the simple effect of time on each treatment individually for the significantly affected parameters, we found that *C_epi_* showed a significant increase over time for the AT treatment (*p* < 0.003), while there was the opposite trend for the NT condition (*p* < 5 × 10^−4^) (Figure 4c). At the electrode level, *R_ct_* decreased in all the conditions, although the change was barely not significant for the μNA condition (*p* = 0.06, whereas AT, SA, and NT: *p* < 0.05, Figure 4e). *C_dl_* only increased significantly for the AT treatment condition (*p* < 4.0 × 10^−4^) (Figure 4f).

For the model parameters that did not show significant time-treatment interaction (i.e., *R_sub_*, *R_epi_*, and *R_gel_*), we assessed only the significance of the main effect of time. At the skin level, the average value of *R_sub_* remained between 0.67–0.81 kΩ cm^2^, and did not change significantly across time or treatment types (Figure 4a). In contrast, the main effect of time was found to be significant for *R_epi_* (F = 16.8, *p* < 2.7 × 10^−7^), with values decreasing over time for all skin conditions (Figure 4b). Finally, at the electrode level, the value of *R_gel_* did not vary significantly over time or treatment type, and remained well within the bounds set by our model (Table 1, Figure 4d).

## 4. Discussion

The goal of the present study was to explore time-dependent impedance changes at the skin–electrode interface as a function of skin pre-treatment over a 24 h period of wear time. Four representative skin treatments were explored, with varying degrees of harshness. The AT treatment is commonly adopted in clinical practice to fully abrade the stratum corneum, and represents the most invasive treatment relative to NT. The SA treatment is a chemical exfoliation method offering gentler abrasion of the stratum corneum. Thirdly, we chose microporation with a µNA to test whether we could create more readily accessible routes to sweat and oils from the deeper tissue layers, allowing faster hydration of the skin surface. Finally, NT is the control condition of no treatment applied to the skin after cleaning with alcohol. We measured the skin–electrode interface impedance at an initial timepoint, then 8 and 24 h later, and modelled the total impedance response with the equivalent circuit model proposed in Figure 2a and described by Equation (1). The model is composed of two RC parallel components representing the skin and electrode, respectively, connected by the resistance of the gelled electrolyte that coats the electrode surface. Thus, this model allows us to identify the separate contributions of the skin and the electrode to charge-transfer at the interface over time.

The plots in Figure 2b,c show that the results of the fitting are in good agreement with the experimental data, demonstrating that the proposed equivalent circuit model—with its bounded parameter space—adequately represents the impedance frequency response under different skin conditions. From the model Equation (1), at high frequencies the total impedance is dominated by the subcutaneous and gelled electrolyte resistances (*R_sub_* and *R_gel_*), while at lower frequencies, the total impedance becomes the sum of all resistive components, with the epidermal and electrode charge-transfer resistances, *R_epi_* and *R_ct_,* respectively, adding to *R_sub_* and *R_gel_*. Thus, while more frequency-independent effects dominate the impedance behavior in the high and low frequency regimes, capacitive attenuation occurs between these extremes. In the higher frequency regime, the skin dominates the impedance behavior, as seen by the similarity between the moduli curves at *f* > 5 kHz (Figure 2b). For the AT treatment, however, the electrode begins to dominate the impedance frequency response around ~4 kHz, resulting in a lower overall impedance that persists into the lower frequency regime. In contrast, for the SA, µNA, and NT treatments, the capacitive response shifts the cutoff frequencies towards lower frequencies. In the frequency ranges relevant for recording biopotentials such as the electroencephalogram (EEG, 0.1–100 Hz), EMG (25–500 Hz), or ECG (0.5–150 Hz) [46], for all the conditions the impedance already shows a predominantly resistive response; however, due to the capacitive attenuation of the skin, in the milder conditions the impedance is higher than in the case of abrasion (Figure 2b,c). Overall these results indicate that, as the skin treatment becomes harsher, the capacitive attenuation from the skin is less relevant and the impedance response becomes progressively more resistive, shifting towards lower magnitude values and higher cutoff frequencies.

It is also worth noting that the trends seen in the overall impedance as it changes over time are expected, given the various treatment types. The milder treatments—NT, SA, and µNA—showed decreases in their 10 Hz impedance from the *t*_0_ to *t_f_* timepoints (Figure 2d–f), which is consistent with the hypothesis that natural skin hydration decreases the interfacial impedance due to the presence of ions in human sweat [12]. Additionally, there was a slight increase in the impedance of the skin under the AT treatment over 24 h. While the change was minimal over time, it may still be explained from a biological standpoint. After abrasive damage, the stratum corneum naturally recovers within 12 h [47], and after 24 h, the uppermost layers of the epidermis are largely repaired. Indeed, the stratum corneum has a natural cycle of self-repair that it completes in order to maintain its normal function as the outermost skin layer and environmental barrier [48].

The trends seen in the overall impedance for each treatment are also reflected in the individual components of the equivalent circuit model at the initial timepoint. Immediately after electrode application, the epidermal components (*R_epi_* and *C_epi_*) are significantly lower for AT compared to all other conditions (Figure 3b,c), while the subcutaneous resistance, *R_sub_*, remains uniform across treatment types. This indicates that impedance improvement is largely due to removal of the superficial epidermal layers, while the deeper skin layers remain undisturbed [4,49,50]. In particular, the stratum corneum is a hydrophobic insulating layer, and is the main barrier to signal transduction between the skin and the electrodes [4,12]. At the electrode level, the charge-transfer resistance, *R_ct_*, is significantly lower under the AT condition compared to the other treatments (Figure 3e), whereas the double-layer capacitance, *C_dl_*, is not significantly different among the different skin conditions (Figure 3f). The lower *R_ct_* value for AT may be explained by the fact that in non-polarizable, faradaic electrodes—such as the Ag/AgCl electrodes used in this study—charge-transfer characteristics are typically dominated by *R_ct_* [19,21,22], and thus interventions at the skin-level should have less effect on *C_dl_* than on *R_ct_*. Finally, and as expected given the similarity between commercially manufactured electrodes, *R_gel_* is comparable across all skin conditions (Figure 3d).

Looking at the time evolution of the model parameters, *R_sub_* does not show significant variations within 24 h (Figure 4a), confirming the validity of the model to capture the largely invariant characteristics of the subdermal layers. At the epidermal level, however, all treatments show an overall ~50% decrease in *R_epi_* over time (Figure 4b, Table 1). This change is likely the result of hydration of the skin via release of sweat under the electrodes, since sweat breaking through the hydrophobic stratum corneum contributes to direct charge-transfer between the skin and the electrode gel. The model also captures an equilibration of *C_epi_* over time, to values in the range of 30–45 nF cm^−2^ among all treatments (Figure 4c, Table 1), even though at *t*_0_ the abrasion appeared to significantly reduce the skin capacitance (Figure 3c). The slight increase seen in the *C_epi_* results for the AT treatment over time are likely related to changes in the dielectric constant of the stratum corneum as it repairs itself [47,51]. The findings of a time-only interaction for *R_epi_* and a time-treatment interaction for *C_epi_* indicate that sweating changes both the capacitive attenuation of the interfacial impedance and the faradaic effects at the skin level. Thus, we can conclude that at the skin level the main effects on different skin treatments over time are changes in the epidermal capacitance (Figure 4b,c) and that abrasion of the superficial layers allows the skin to retain the predominantly faradaic behavior observed at *t*_0_.

At the electrode level, *R_gel_* appears to be invariant with time or treatment. While it is known that gelled electrodes dry out over time, 24 h may not be nearly enough time to observe this phenomenon [52]. However, for the three milder skin treatments, *R_ct_* decreases dramatically between *t*_0_ and *t_mid_*, and equilibrates by *t_f_* (Figure 4e), whereas *C_dl_* remains essentially unchanged (Figure 4f). Comparatively, electrodes in the AT condition show an overall decrease in *R_ct_* and a slight increase in *C_dl_* by *t_f_.* These changes appear to be significant also from a time-treatment interaction standpoint (Table 2), highlighting that evolution in the electrode-level parameter values also depends on the type of skin treatment used before electrode placement. The variability of *R_ct_* in particular across all treatment types underscores the point that the majority of charge-transfer through gelled electrodes, even over time, occurs through this specific component more so than through the *C_dl_* component of the electrode branch. Again, this is expected, due to the non-polarizable nature of gelled Ag/AgCl electrodes [17].

Altogether, these results indicate that for gelled electrodes, the impedance is largely controlled by the epidermis. Thus, after skin treatment and electrode placement, there is no significant difference between NT and an intervention that does not effectively remove the stratum corneum. Since *R_epi_* for the SA and µNA interventions were comparable to the NT condition at *t*_0_ (Figure 3b,c), we can conclude that these interventions are not sufficient to overcome the epidermal barrier and facilitate direct charge-transfer. Only mechanical abrasion seems to effectively reduce *R_epi_* and *C_epi_*. Our analysis also reveals that the initial skin conditions have an important effect on the electrode-skin impedance in the short-term, but that over time these differences tend to reduce. By the final timepoint, although the AT condition still had the lowest impedance compared to the other treatments, all conditions showed total impedance moduli that were < 20 kΩ at 10 Hz. This time evolution of the skin–electrode response is likely driven by natural skin hydration, and results in all skin conditions adjusting to reach equilibrium within the explored 24 h timeframe.

## 5. Conclusions

In this work, we have proposed an experimental and modeling framework to evaluate the interfacial impedance at acute and chronic timepoints. The framework is based on an equivalent circuit model with separate skin and electrode branches, allowing us to investigate the specific contributions of each level to the total impedance response, as well as the effects of interventions on the properties of each branch. To illustrate the applicability of this approach in a typical use-case scenario, we applied the equivalent circuit model to pre-gelled Ag/AgCl electrodes interfacing with the skin, and evaluated the effects of four different skin conditions on the impedance over 24 h. The results of our analysis suggest that skin abrasion is the most effective at lowering the skin impedance because it removes the insulating barrier presented by the epidermis, thus favoring direct charge-transfer between the skin, gel, and electrode. Alternative skin interventions that likewise remove or decrease the epidermal insulating barrier may also offer benefits to the interfacial impedance, such as controlled laser microporation [53] or electroporation [54]. However, likely due to sweating and natural skin hydration, after only 8 h the impedance may still be sufficiently low (≈20 kΩ at 10 Hz) to enable low-noise recordings, even without any skin treatment. The equilibration of the skin–electrode impedance response appears to be primarily driven by changes in the faradaic elements, both at the skin and electrode levels. An improved understanding of the skin–electrode interface, and the ability to discriminate the different contributions to the overall impedance as proposed in this work, may help in the development of alternative skin treatment interventions. Furthermore, with the growing interest in gel-free electrodes [38,39,55] and in innovative gelled electrode technologies [56,57], a rigorous understanding of the factors affecting the skin–electrode impedance can enable improved design of high-fidelity biosensors for future wearable applications, both in the clinical and consumer markets. Finally, with the proposed model, it will be possible to more efficiently recognize and track variations in the impedance of skin-based electrodes over time, which will greatly benefit long-term monitoring and at-home ambulatory applications of wearable biosensing technologies.

## Figures and Tables

**Table 1:** Equivalent circuit elements, their boundaries, and average fitted values for each skin treatment, at *t_0,_ t_mid_,* and *t_f_*. The range for each parameter was determined based on values in literature [4,17], as well as considering the total impedance (see Equation (1) and Figure 2b). Values are reported as avg. ± std. dev. across *N* = 14 subjects.

		*R_sub_* [kΩ cm^2^]	*R_epi_* [kΩ cm^2^]	*C_epi_* [nF cm^−2^]	*R_gel_* [kΩ cm^2^]	*R_ct_* [kΩ cm^2^]	*C_dl_* [nF cm^−2^]	Model Fit $\chi^{2}$ (×10^2^)
**Lower-Upper** **Parameter bounds**		0.05–5.00	0.10–500	0.10–500	0.05–5.00	0.5–5.0 × 10^3^	1.0–1.0 × 10^5^	—
**No Treatment**	*t*_0_	0.71 ± 0.16	86.38 ± 29.43	242.62 ± 148.41	0.87 ± 0.46	(1.31 ± 1.27) × 10^3^	5.38 ± 2.19	3.75 ± 0.71
	*t_mid_*	0.74 ± 0.19	47.07 ± 33.09	27.98 ± 26.86	1.07 ± 0.90	147.93 ± 101.27	63.42 ± 25.62	2.18 ± 1.85
	*t_f_*	0.71 ± 0.20	40.90 ± 30.70	34.70 ± 19.11	0.72 ± 0.42	66.54 ± 31.96	36.29 ± 15.24	3.09 ± 0.84
**Abrasive Tape**	*t*_0_	0.70 ± 0.20	6.61 ± 5.02	4.95 ± 3.73	0.59 ± 0.13	2.90 ± 2.39	7.00 ± 2.90	2.97 ± 0.75
	*t_mid_*	0.72 ± 0.18	1.80 ± 1.45	8.49 ± 2.46	0.59 ± 0.11	3.30 ± 2.66	10.50 ± 6.56	0.27 ± 0.08
	*t_f_*	0.67 ± 0.22	2.27 ± 1.39	28.28 ± 20.65	0.76 ± 0.48	1.26 ± 0.49	12.23 ± 4.27	0.36 ± 0.18
**Salicylic Acid**	*t*_0_	0.79 ± 0.14	69.43 ± 30.09	97.63 ± 76.73	0.65 ± 0.14	675.80 ± 501.10	9.97 ± 5.40	4.72 ± 0.97
	*t_mid_*	0.77 ± 0.18	48.39 ± 32.86	47.81 ± 27.09	1.17 ± 1.02	17.67 ± 14.97	9.88 ± 9.54	0.97 ± 0.05
	*t_f_*	0.77 ± 0.16	35.97 ± 33.36	44.74 ± 31.84	1.36 ± 1.18	12.76 ± 9.71	7.70 ± 3.40	1.15 ± 0.49
**µNeedle Array**	*t*_0_	0.81 ± 0.14	81.56 ± 31.34	29.46 ± 22.32	0.67 ± 0.17	807.42 ± 394.97	5.05 ± 2.17	4.15 ± 1.40
	*t_mid_*	0.74 ± 0.18	46.50 ± 35.41	24.59 ± 21.80	1.19 ± 1.09	92.16 ± 60.74	9.50 ± 5.52	2.62 ± 1.34
	*t_f_*	0.71 ± 0.16	35.36 ± 32.01	33.65 ± 28.34	0.91 ± 0.67	72.29 ± 30.89	5.38 ± 2.19	1.22 ± 0.40

**Table 2:** Results of likelihood ratio testing (LRT) for the equivalent circuit model parameters. The significance level was set to *p* = 0.05.

Element	Interaction Effect $\chi^{2}$(*df* = 6) (*p*-Value)	Main Effect F(*df* = 2) (*p*-Value)	Simple Effect F(*df* = 2) (*p*-Value)			
			NT	AT	SA	µNA
***R_sub_***	3.0 (0.81)	0.9 (0.4)	--	--	--	--
***R_epi_***	8.8 (0.19)	16.8 (2.7 × 10^−7^)	--	--	--	--
***C_epi_***	34.2 (6.13 × 10^−6^)	--	9.5 (4.6 × 10^−4^)	7.0 (0.003)	0.9 (0.4)	2.1 (0.1)
***R_gel_***	6.0 (0.43)	0.8 (0.4)	--	--	--	--
***R_ct_***	58.0 (1.16 × 10^−10^)	--	30.2 (1.4 × 10^−8^)	3.7 (0.03)	21.2 (5.9 × 10^−7^)	3.1 (0.06)
***C_dl_***	58.0 (1.15 × 10^−8^)	--	1.0 (0.4)	10.0 (3.9 × 10^−4^)	1.2 (0.32)	1.9 (0.16)

## Data Availability

Please contact the corresponding author with questions regarding data sharing and availability.
